# Effect of Treatment Modality on Long-Term Outcomes in Attention-Deficit/Hyperactivity Disorder: A Systematic Review

**DOI:** 10.1371/journal.pone.0116407

**Published:** 2015-02-25

**Authors:** L. Eugene Arnold, Paul Hodgkins, Hervé Caci, Jennifer Kahle, Susan Young

**Affiliations:** 1 Research Unit on Pediatric Psychopharmacology, The Ohio State University, Columbus, Ohio, United States of America; 2 Global Health Economics & Outcomes Research, Shire, Wayne, PA, United States of America; 3 Hôpitaux Pédiatriques de Nice CHU Lenval, F-06200, Nice, France; 4 BPS International, San Diego, CA, 92130, United States of America; 5 Imperial College London, Centre for Mental Health, London, SW7 2AZ, United Kingdom; University of Frankfurt, GERMANY

## Abstract

**Background:**

Evaluation of treatments for attention-deficit/hyperactivity disorder (ADHD) previously focused on symptom control, but attention has shifted to functional outcomes. The effect of different ADHD treatment periods and modalities (pharmacological, non-pharmacological, and combination) on long-term outcomes needs to be more comprehensively understood.

**Methods:**

A systematic search of 12 literature databases using Cochrane’s guidelines yielded 403 English-language peer-reviewed, primary studies reporting long-term outcomes (≥2 years). We evaluated relative effects of treatment modalities and durations and effect sizes of outcomes reported as statistically significantly improved with treatment.

**Results:**

The highest proportion of improved outcomes was reported with combination treatment (83% of outcomes). Among significantly improved outcomes, the largest effect sizes were found for combination treatment. The greatest improvements were associated with academic, self-esteem, or social function outcomes. A majority of outcomes improved regardless of age of treatment initiation (60%–75%) or treatment duration (62%–72%). Studies with short treatment duration had shorter follow-up times (mean 3.2 years total study length) than those with longer treatment durations (mean 7.1 years total study length). Studies with follow-up times <3 years reported benefit with treatment for 93% of outcomes, whereas those with follow-up times ≥3 years reported treatment benefit for 57% of outcomes. Post-hoc analysis indicated that this result was related to the measurement of outcomes at longer periods (3.2 versus 0.4 years) after treatment cessation in studies with longer total study length.

**Conclusions:**

While the majority of long-term outcomes of ADHD improve with all treatment modalities, the combination of pharmacological and non-pharmacological treatment was most consistently associated with improved long-term outcomes and large effect sizes. Older treatment initiation age or longer durations did not markedly affect proportion of improved outcomes reported, but measurement of outcomes long periods after treatment cessation may attenuate results.

## Introduction

There is increased global recognition that attention-deficit/hyperactivity disorder (ADHD) is a serious and common neurodevelopmental condition that can persist over the lifespan. To meet diagnostic criteria, the symptoms of ADHD must result in impairment in functional domains across multiple settings; thus, impairing and enduring symptoms lead to a lower health-related quality of life [1–3]. Adults diagnosed with ADHD in childhood, but not treated for ADHD, have been reported to have poor outcomes in a wide range of areas of life, including social function, education, criminality, alcohol use, substance use, and occupational outcomes [4,5], and declines in outcomes over time have been reported with untreated ADHD for drug use [6] and academic outcomes [7–9].

Research is now taking a lifespan perspective of the outcomes of ADHD, as studies of prevalence of ADHD continue to confirm the global presence of ADHD in children, adolescents, and adults. Meta-analyses of 86–102 studies estimated a worldwide point prevalence of 5.3–7.1% in children and adolescents [10,11]. Adult ADHD prevalence in the United States has been estimated at 4.4% [12] and 1.2–7.3% among several countries world-wide [13–15]. Consolidation of data from studies of all ages from countries across the globe [3] showed that untreated ADHD is commonly associated with poor long-term outcomes in a wide range of areas that affect the patients, their families, and society in general.

Although treatment of ADHD has traditionally focused on symptom control, patient management has evolved in recent years to include greater focus on functional impairment and outcomes [16]. Shaw et al [3] demonstrated that treatment of ADHD was often associated with improved long-term outcomes compared with untreated ADHD. There remain, however, several questions regarding specific aspects of treatment and for which there has yet to be a synthesis of research findings. Of primary interest is the comparison of benefit of different ADHD treatment modalities (pharmacological, non-pharmacological, or a combination of the two) to long-term outcomes of ADHD [16]. Using an approach similar to Shaw et al. [3], we therefore conducted additional analysis to examine the primary research question: Are there differences in treatment benefit to long-term outcomes among the different types of treatment (pharmacological, non-pharmacological, or combination treatment)? In addition, two secondary questions were posed: Does age of initiation of treatment have an effect on long-term outcomes [17]? Does treatment duration affect benefit to ADHD outcomes [18]? Answers to these questions become important at a time when treatment practices are changing in response to the widening ages at which ADHD diagnoses are being made [19–21]. Age of treatment initiation is important because with the refinements of later onset ADHD and adult ADHD in the newest Diagnostic and Statistical Manual of Mental Disorders (DSM-5) [19], there will be a patient population starting treatment later in life compared with the historical early-school-years treatment starters. Furthermore, it has been estimated that for a significant proportion, perhaps half of childhood-diagnosed patients, ADHD persists into adulthood [19]. It is important to examine the ramifications of a longer duration of treatment as patients who were medicated in childhood also may consider extending their treatment into adulthood.

## Methods

### Identification of studies

**Fig 1:**
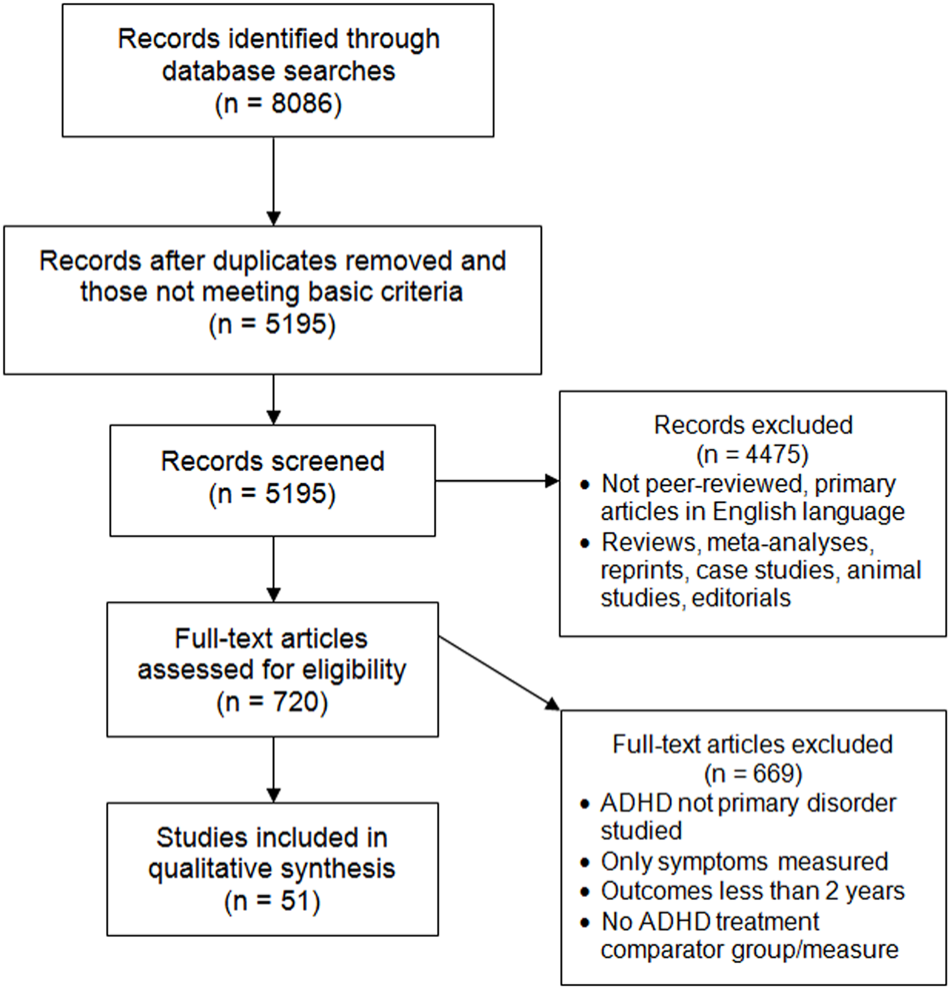
Flow diagram showing the selection process and results during the study screening process.

An extensive and systematic search was conducted of 12 literature databases to identify peer-reviewed, primary studies that reported long-term outcomes of individuals with ADHD. The databases were Academic Search Premier, CINAHL, Cochrane CRCT (including EMBASE), Criminal Justice Abstracts, ERIC, MEDLINE, Military & Government collection, NHS Economic Evaluation database, PsycARTICLES, PsycINFO, SocINDEX, and Teacher Reference Center. All 12 databases were searched in two search waves conducted in May 2010 and March 2011. A third search of 9 databases was conducted in March 2012. Three databases were not utilized in the third search (Academic Search Premier, NHS Economic Evaluation database, and PsycARTICLES) because no unique useful citations had been derived from those databases in the first searches. All other methods were held constant between the 3 search waves (see S1 Appendix for search string and limits). Search limits included English-language and publication date from January 1980 through December 2011 inclusive. Duplicates were eliminated electronically and manually. Based primarily on title and abstract, these studies were reviewed manually and inclusion was agreed on by two researchers. All disagreements between researchers on study inclusion were resolved by examining the full text of the study. Inclusion required that studies be published as peer-reviewed, primary research articles in the English language with full text available. Inclusion also required that the study had a comparator group (e.g., individuals with untreated ADHD) or a comparison measure (e.g., pre-treatment baseline), and that ADHD was a primary disorder under study. Treatments included pharmacological, non-pharmacological, or combination treatments/interventions intended for treatment of ADHD. Only studies reporting long-term outcomes of 2 years or more (follow-up time, not necessarily treatment duration) were included. The 2-year long-term outcome criterion could be met by longitudinal studies with prospective follow-up measurements ≥2 years, retrospective studies with a time period ≥2 years, cross-sectional studies comparing two ages differing by 2 years or more, or cross-sectional studies of individuals age 10 years or older. Age 10 was chosen as the minimum age for single-age, cross-sectional studies, based on the Diagnostic and Statistical Manual of Mental Disorders, Fourth Edition, Text Revision (DSM-IV-TR) diagnosis criteria that symptoms be present before age 7 years, thus conservatively allowing at least 2 years to pass before outcomes were measured. Meta-analyses, case studies, and literature reviews were excluded. A checklist of Preferred Reporting Items for Systematic Reviews and Meta-analyses (PRISMA) is provided in S2 Appendix and a PRISMA flow diagram of the study selection process [22] is provided in Fig. 1.

### Data extraction

Reported data from each study was manually extracted from the full text of the study to a database, including: (1) study location, (2) study sample size, (3) study length (e.g., years to follow-up), (4) participant sex and age range, (5) diagnostic criteria or symptom assessment, (6) study type (longitudinal, cross-sectional, prospective, retrospective, randomized), (7) outcome results, (8) comparator type, (9) treatment type, (10) treatment initiation age, and (11) treatment duration.

### Evaluation of outcome measures

All reported long-term outcomes were organized into 9 main categories/domains based on common characteristics: 1) academic (e.g., achievement test scores, grades, length of education, repeated grades, education level), 2) antisocial behavior (e.g., school expulsion, delinquency, police contacts, arrests, convictions, incarceration, self-reported crimes, types or severity of offenses, age at first incident, repeat convictions), 3) driving (e.g., traffic violations, automobile accidents, license status, driving simulation rating), 4) non-medicinal drug use/addictive behavior (e.g., substance use, abuse, and/or dependence—from caffeine to illicit drugs; age at initiation; quitting substance use; amount of substance used; non-substance addictions such as gambling), 5) obesity (body mass index, weight), 6) occupation (e.g., employment, military service, income/debt, job performance, job loss/changes, occupation level, socioeconomic status), 7) services use (e.g., school services, health services, emergency room visits, work-related services, financial assistance, justice system), 8) self-esteem (self-esteem questionnaires, suicide ideation, suicide attempts, suicide rate), and 9) social function (e.g., peer, family, and romantic relationships; peer nomination scores; marital status; divorce rate, social skills, living arrangements, activities/hobbies). Note that ADHD symptoms were not considered to be a type of long-term outcome.

Except in the analysis of individual effect sizes, the individual measures reported in each study were summarized as a single “improved” or “no-benefit” outcome for each outcome category. Clinical significance was rarely reported, so an outcome was considered “improved” with treatment when a measure was reported to have statistically significant improvement associated with treatment compared with individuals with untreated ADHD or pre-treatment baseline. An outcome was considered to have “no benefit” with treatment when measures were either poorer or not statistically significantly different compared with pre-treatment baseline or individuals with untreated ADHD. Proportions of improved and no-benefit outcomes reported were then compared non-statistically. A risk of Type 2 error must be acknowledged for some studies (e.g., some differences may not have reached statistical significance due to small sample size).

The effect sizes of improved outcomes associated with pharmacological, non-pharmacological, or combination treatment were compared through analysis of measures that were reported to be statistically significantly improved with treatment, and either the effect size was reported or sufficient information was reported so that effect size could be calculated. Cohen’s *d* was derived from continuous measures for which results were reported as means (± standard deviation), such as scores on a math achievement test. Cohen’s *w* was derived from discrete measures of incidence, such as number of people who smoked cigarettes last month, often derived from the related reported χ^2^ (chi squared) statistic. Cohen’s *f* was derived from results of analysis of variance (ANOVA) or regression analyses reported as the η^2^ or η (eta squared or eta) statistic, such as the likelihood of cocaine use related to stimulant medication history controlling for the presence of conduct disorder. Effect size derivation was according to methods in Cohen, 1988 [23]: The difference in means divided by the pooled standard deviation of the means for Cohen’s *d*, square root of reported χ^2^ divided by the study sample or from a 2×2 contingency table for Cohen’s *w*, and Cohen’s *f* was converted from reported η^2^ (or η) using conversion tables in Cohen, 1988 [23]. Effect sizes were evaluated according to Cohen’s conventional criteria for small, medium, and large effect sizes (Cohen’s *d*: 0.2, 0.5, 0.8; Cohen’s *f*: 0.1, 0.25, 0.4; Cohen’s *w*: 0.1, 0.3, 0.5, respectively).

For each of the nine outcome categories, the proportion of studies of each treatment modality (pharmacological, non-pharmacological, and combination treatment) that reported significant improvement was analyzed. The effect of treatment duration was examined by dividing studies into those with shorter treatment duration (mid-range treatment of individuals ≤2 years) or longer treatment duration (mid-range >2 years) and determining the proportion of studies demonstrating improvement or no benefit on outcomes with treatments from either group. Treatment duration of 2 years was chosen for this analysis to correspond with several previous studies of treatment duration of 2 years or less [24–31]. Effect of participant age at initiation of treatment was analyzed by dividing studies into three groups according to mid-range age at initiation of treatment (<8 years old, 8–12 years old, and ≥13 years old) and determining the proportion of studies demonstrating benefit or no benefit for each initiation age category. These age category divisions were chosen to correspond with the minimum age of adolescence used in this analysis for other age categorizations (13 years) and to provide a sample of the youngest age-of-initiation studies available in this dataset (<8 years old). The influence of several aspects of study design and participant characteristics was also examined. Outcomes reported in studies of specific study designs (e.g., randomized treatment studies) were examined. The effect of total study length (i.e., time to follow-up measurement <3 years versus ≥3 years) was examined. The effect of participant sex and age at follow-up were examined. Age at follow-up was also analyzed for each outcome domain by categorizing study groups by mid-range age at the longest follow-up time as children, (6–12 years old), adolescents (13–17 years old), or adults (18–64 years old), regardless of age at treatment initiation or treatment duration.

## Results

### Effect of different treatment modalities on ADHD outcomes

There were 51 studies (111 outcomes total; see S3 Appendix for complete list of citations) that reported outcomes associated with ADHD treatment in comparison with no treatment for ADHD (either pre-treatment baseline or an untreated group with ADHD). There were more outcomes than studies because many studies reported more than one outcome. The effect of different treatment modalities (pharmacological, non-pharmacological, and combination treatment) was examined (see S4 Appendix for a complete list of specific treatments included in the studies). Table 1 shows the proportion of outcomes reported to improve significantly or not with the three different ADHD treatment modalities. The majority of the reported outcomes (56%, 62/111) were from studies of the effects of pharmacological treatment, and 50% (31/62) of these outcomes were reported to exhibit significant improvement. For non-pharmacological treatment, 65% (17/26) of the outcomes were reported to exhibit significant improvement. The highest proportion of outcomes exhibiting significant improvement was reported for combination treatment, 83% (19/23).

**Table 1:** Proportion of outcomes with improvement or no benefit with three different ADHD treatment modalities.

	Improvement			No significant improvement		
	# outcomes	% of treatment type	% of improved outcomes	# outcomes	% of treatment type	% of “no benefit” outcomes
**Pharmacological**	31	50%	46%	31	50%	70%
**Non-pharmacological**	17	65%	25%	9	35%	20%
**Combination**	19	83%	28%	4	17%	9%
**All treatment types**	67	60% of total		44	40% of total	

### Degree of improvement; effect sizes

**Fig 2:**
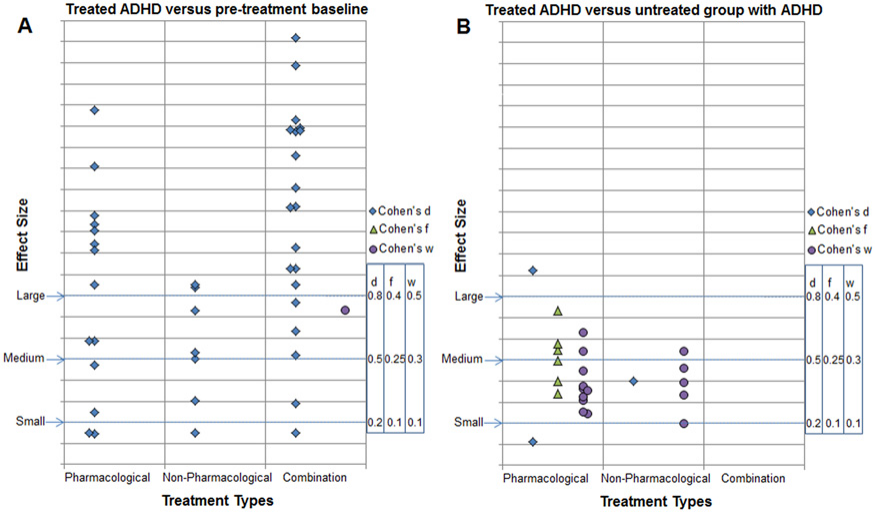
Effect Sizes. Effect sizes for outcomes reported to have statistically significant improvement with treatment compared with pre-treatment baseline scores (A) or a group of individuals with untreated ADHD (B). Effect sizes were grouped according to treatment modality used in each study and graphed with Y-axis scales appropriate for each index (Cohen’s *d*, *f*, or *w*) that were then matched at conventional criteria [23] for small, medium, and large effect sizes (dashed lines) for comparison (values for each index shown on right Y-axis).

The degree of improvement with treatment for those outcomes that were statistically significantly improved was expressed as effect sizes that were then grouped by comparator type, treatment modality, and effect size index (*d*, *w*, or *f*) for comparison (Fig. 2). All effect sizes that were reported or could be derived for statistically significant improvements are included (numerical listing in S5 Appendix).

Effect sizes ranged from below the small threshold to above the large threshold (Fig. 2A,B). All but one of the effect sizes falling above the large threshold were for outcomes within the academic, self-esteem, or social function domains, suggesting that these outcomes exhibited the greatest improvements associated with treatment. Note that these effect sizes are only for outcomes reported to be statistically significantly improved.

### Effect of treatment modality on each outcome domain

**Fig 3:**
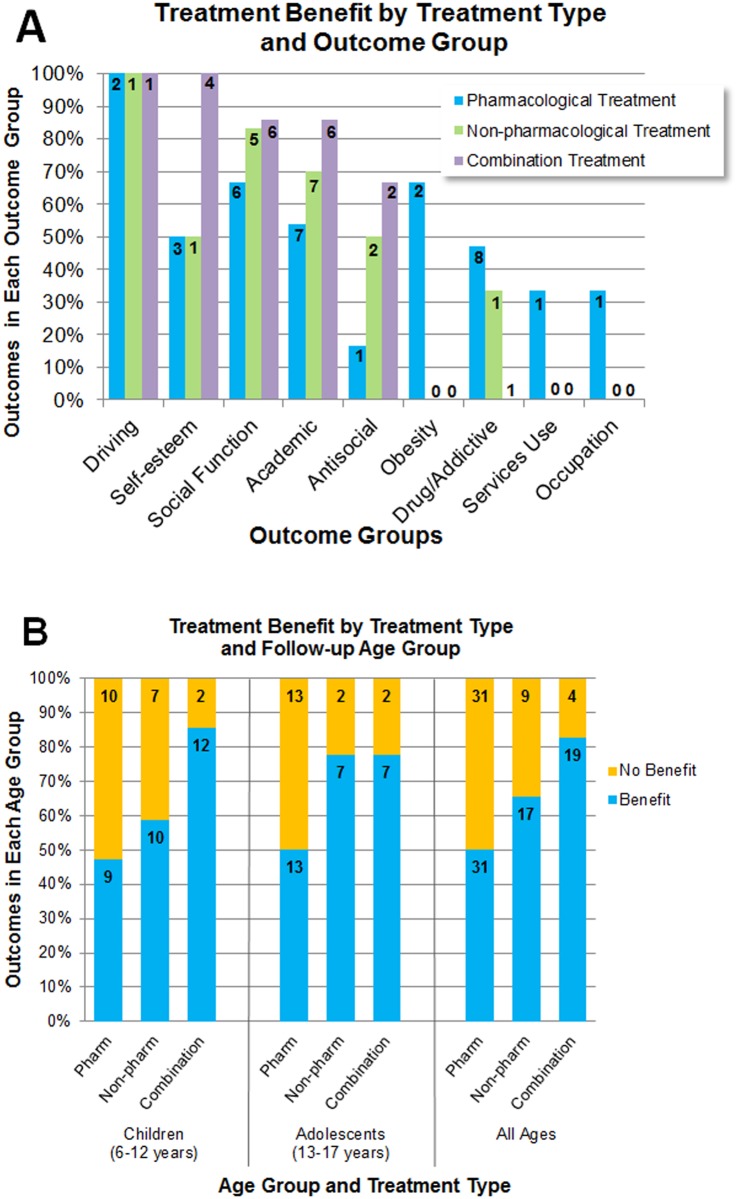
Treatment Modalities. (A) Benefit with each treatment modality for each outcome domain. Each bar represents the percentage of outcomes reported to exhibit benefit (either significantly improved from untreated baseline or significantly improved compared with a group of untreated individuals with ADHD) with each treatment modality. (B) Benefit with each treatment modality for different age groups at follow-up. The colored sections within bars represent the percentage of outcomes reported to improve (benefit) or not (no benefit) for each treatment modality for children (mid-range age 6–12 years) and adolescents (mid-range age 13–17 years), and all ages. Adults (mid-range age 18–84 years) are not presented separately because there were only studies of pharmacological treatment in this age group at follow-up. The numbers on the bars indicate the number of outcomes represented in each bar. Some studies reported outcomes with more than one type of treatment.

Separating the results by type of outcome allowed examination of which treatment types were most commonly associated with improvement for each outcome (Fig. 3A). The proportion of outcomes that improved varied among treatment modalities, as did the number of available supporting studies and outcomes. Of the outcomes most amenable to treatment, all treatment modalities were reported to be associated with improvement. For example, pharmacological, non-pharmacological, and combination treatments all contributed to the 100% of studies reporting improved outcomes for driving. Similarly, a high percentage of beneficial results for social function outcomes were reported with all three treatment modalities: pharmacological (67%, 6/9), non-pharmacological (83%, 5/6), and combination treatment (86%, 6/7). All treatment modalities also contributed to improved outcomes in the self-esteem, academic, and antisocial behavior domains.

The number of studies meeting inclusion criteria was limited for some outcome domains and treatment modalities. For example, in the domain of drug use/addictive behavior outcomes, effects of pharmacological treatment represented most of the studies (81%, 17/21) with 47% (8/17) of outcomes exhibiting improvement. Non-pharmacological treatment was assessed in only 3 drug-use/addictive behavior studies and was associated with improved outcomes in only 33% (1/3) of outcomes reported. Combination treatment for drug use/addictive behavior outcomes was evaluated in one study and was not significantly associated with improvement. For self-esteem, social function, academic, and antisocial behavior outcomes, a greater proportion of outcomes following combination treatment showed improvement compared with outcomes following pharmacological or non-pharmacological treatment. In the cases of obesity, services use, and occupation outcomes, only studies with pharmacological treatment were identified, so treatment modalities could not be compared.

Considering that obesity, services use, and occupation outcomes were not reported for all three treatment modalities, we re-analyzed the overall improved outcomes of the three treatment modalities without these outcomes included. This sensitivity analysis examined outcomes measured for all three treatment modalities (102 total outcomes), to reduce possible bias of association between treatment modality and outcome type. The results were similar to those presented in Table 1 with all outcomes included, with percentages changing by 1–3 percentage points. The proportion of significantly improved pharmacological treatment outcomes increased from 50% to 51%.

### Effects of each treatment modality for different age groups at follow-up

The proportion of outcomes that improved with each treatment type examined in children and adolescents at follow-up were similar to results in all age groups (Fig. 3B), and these results did not support any strong age-group by treatment-modality interaction. Adults were not presented separately in this analysis because only pharmacological treatment was assessed in studies of adults. Approximately half (47–53%) of the outcomes were reported to improve with pharmacological treatment for each age group, including adults. A high percentage of outcomes were improved with combination treatment, measured only in children or adolescents (78–86%).

### Effect of age of treatment initiation on outcomes

The age at which therapy is initiated may be an important factor influencing long-term outcomes. Of the total 51 studies reporting treatment effects compared with untreated ADHD, 40 studies reported the age of initiation of treatment, often as a range. These studies were divided into three groups according to mid-range age at initiation of treatment (<8 years old, 8 to 12 years old, and ≥13 years old).

**Fig 4:**
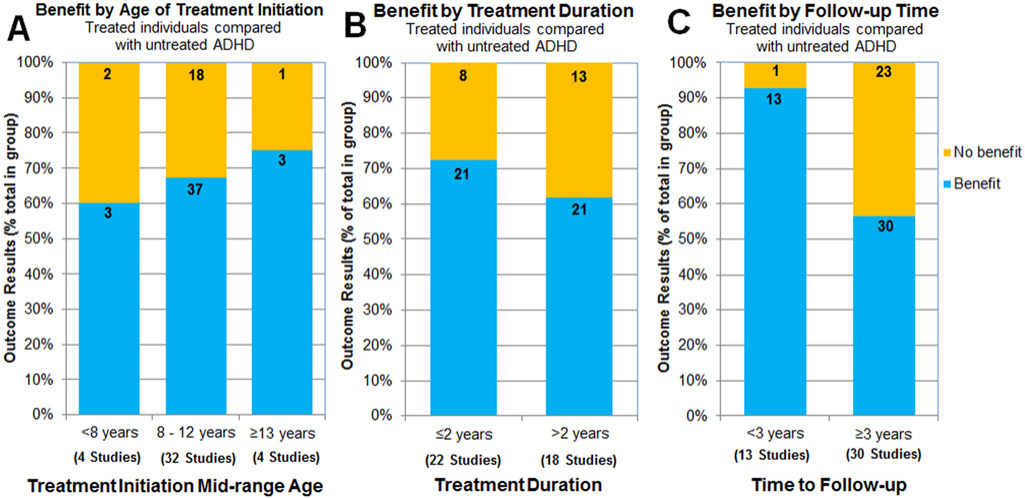
Effect of age of treatment initiation, treatment duration, and follow-up time. Bars represent the percentage of outcomes reported for each age-of-initiation group (A), treatment duration (B), or time to follow-up measures (C). The numbers on the bars are the number of outcomes represented by the section of the bar. The number of studies represented in each bar is presented below the bars.

A high proportion of outcomes (60–75%) were reported to improve with treatment regardless of age of treatment initiation (Fig. 4A). Most of the studies (80%, 32/40) described findings with age of initiation of 8 through 12 years old. Findings with this age-of-initiation group included all treatment modalities and all types of outcomes. Although there was a tendency for the proportion of beneficial outcomes to increase with age of treatment initiation, a majority of outcomes benefitted with treatment (all modalities represented) even with the younger ages of treatment initiation and encompassed outcome domains of self-esteem, social function, and academics (4 studies). Treatment benefits with the older ages of treatment initiation included obesity and drug use outcomes (4 studies). Of note, studies with younger ages of treatment initiation also had younger ages of follow up, which necessarily limited the type of outcomes measured (e.g., neither driving nor occupation) [3].

### Effect of treatment duration on outcomes

Treatment duration was reported in 40 of 51 studies comparing treated ADHD to untreated ADHD. Depending upon the study design, a range of treatment durations may be reported in one study. Of note, presence or absence of treatment and type of treatment utilized during the intervening time to follow-up were often not monitored in the studies making up this analysis; only treatment durations reported in a study were used in the present analysis. To examine the effect of treatment duration, studies were divided into two groups according to the mid-range duration of treatment (≤2 years or >2 years). Studies of both treatment-duration groups reported a high proportion of outcomes with beneficial effects: 72% (21/29) for ≤2 years treatment duration and 62% (21/34) for treatment duration >2 years (Fig. 4B). A majority (53–75%) of each of the three age-of-treatment-initiation categories were studies of shorter treatment duration (≤2 years).

### Effect of follow-up time on outcomes

The follow-up time considered here is the time from study start until when the last follow-up measurement was taken, regardless of treatment duration or time from end of treatment (lag time). The follow-up times of all 51 studies reporting treatment outcomes ranged from 2 to 13 years. To examine the influence of time-to-follow-up measurement [16,27,32–34], we categorized the studies by follow-up time (<3 years versus ≥3 years). Fig. 4C shows the proportion of outcomes demonstrating improvement or no benefit associated with treatment for each of these follow-up time categories. Both follow-up time categories were associated with a high proportion of outcome improvements, however, the percentage of outcomes reported to improve with treatment was greater with the shorter follow-up time (93%) than with the longer follow-up time (57%) as shown in 13 and 30 studies, respectively.

Considering age-of-treatment-initiation (Fig. 4A), treatment duration (Fig. 4B), and time to follow-up (Fig. 4C), the greatest difference in proportion of outcomes reported to have treatment benefit was observed with shorter versus longer time to follow-up. In a post-hoc analysis of the difference observed with shorter follow-up times, two relationships were noted. Studies with shorter treatment duration (≤2 years) also had a shorter mean time to follow-up measurement (mean 3.2 years compared with 7.1 years for studies with longer treatment duration). We also examined the possibility that the lag time (time between end of treatment and time of follow-up measurement) may explain the greater proportion of treatment benefit with shorter follow-up times. Lag time was estimated for 37 studies by subtracting the duration of treatment from the follow-up time. This estimate was made with the assumption that treatment started at the start of the study. For studies in which treatment started at some point before the study start, lag time was underestimated. The estimated lag time (mean = 0.4 years) was markedly shorter for studies with short <3 year follow-up times compared with studies with long ≥3 year follow-up times (mean lag time = 3.2 years). This indicates that studies with shorter treatment duration (and coincidentally shorter follow-up time) had follow-up assessment closer to the cessation of treatment.

### Study design and study participant characteristics influence on treatment outcomes

**Influence of study design**

Results were consistent among the different study designs observed in the included studies. Because there may be differences in the proportion or magnitude of improvement from pre-treatment baseline versus difference from an untreated group of individuals with ADHD, we compared results with these two comparators. Individuals with treated ADHD were compared with either pre-treatment baseline (n = 44 outcomes) or individuals with untreated ADHD (n = 67 outcomes). Treatment of ADHD resulted in improved outcomes for the majority of outcomes reported for both comparator types: 64% (28/44) of outcomes compared with pre-treatment baseline, and 58% (39/67) compared with untreated individuals with ADHD.

There were 5 randomized controlled trials (RCTs), 41 prospective studies (reporting 65 outcomes), 7 retrospective studies (reporting 14 outcomes), and 3 cross-sectional studies (reporting 3 outcomes). RCTs are often considered the gold standard of study designs testing treatment efficacy. The 7 retrospective studies utilized several sources of historical data. Four studies utilized self-report questionnaires or clinical interview of the patients; three studies accessed medical records or health claims databases; and one accessed criminal records. While most included studies were observational open-label studies, in the 5 RCT studies, individuals were randomized to receive non-pharmacological treatment or no treatment: four (57%) of the 7 total outcomes were reported to improve with treatment. The results from studies grouped by other aspects of study design (all treatment modalities considered) are as follows: 66% of outcomes in the prospective studies improved with treatment, 43% of outcomes improved with treatment in the retrospective studies, and one of three outcomes improved with treatment in the cross-sectional studies.

**Influence of participant sex on treatment outcomes**

A total of 12 studies reported treatment benefit/no benefit separated by the sex of the individuals (n = 4) or by including only one sex in the study (n = 8). Of 25 outcomes reported, 20 were in studies of males only, none in females only, and 5 in studies that reported results for males and females separately. In the 4 studies that compared influence of sex directly, only one reported a sex difference. Drug abuse/addictive behavior outcomes for males treated pharmacologically (primarily stimulants) showed a statistically significant but clinically small (Cohen’s *w* = 0.17) benefit compared with untreated males with ADHD, while females showed neither statistically nor clinically significant benefit (Cohen’s *w* = 0.06). It is difficult to make any general sex comparisons based on the studies restricted to a single sex, because there were no studies with only females. Nevertheless, in the 8 studies restricted to males, the percent outcomes showing benefit was 48% (10/21 total outcomes). While benefit was reported for all treatment modalities, combination treatment showed benefit most consistently.

**Influence of study participant age group at follow-up**

**Fig 5:**
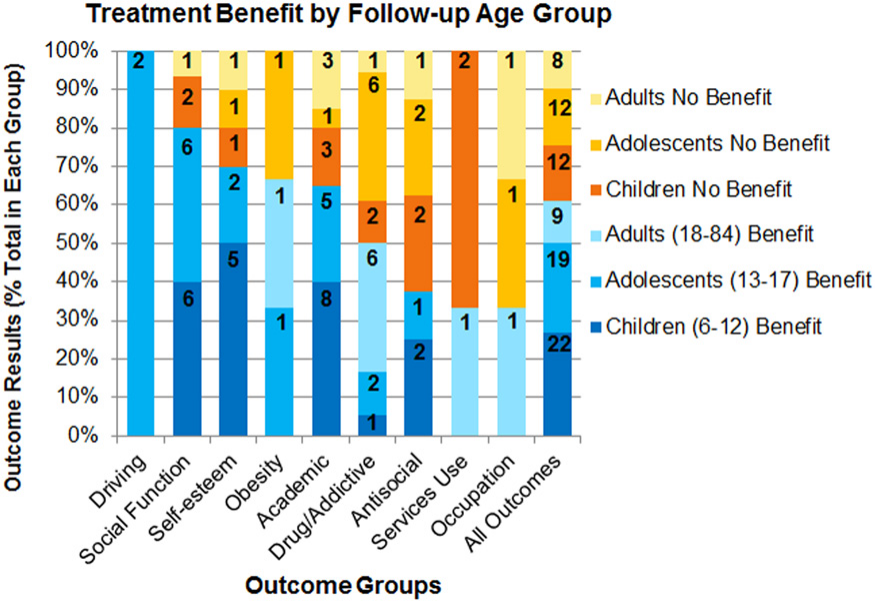
Treatment benefit by follow-up age group for each outcome domain. Colors and shades within bars represent the percentage of outcomes reported for each outcome domain. Blue = benefit; orange-yellow = no benefit. Darker shades indicate younger age groups. The numbers on the bars are the number of outcomes represented by the section of the bar.

Age at which follow-up measures are taken may also be a factor in the reported outcomes of treatment. Considering all outcomes together (far right bar, Fig. 5), there was a higher proportion of improved outcomes versus no benefit within each age group at follow-up: children (65%, 22/34 outcomes), adolescents (61%, 19/31 outcomes), and adults (53%, 9/17 outcomes). Benefit was associated with all treatment modalities in children and adolescents. Only pharmacological treatment was assessed in studies in which follow-up measures were taken in adulthood. Only for drug use/addicitive behavior outcomes were there studies of all age groups at follow-up that reported benefit with treatment. Of note, drug use/addictive behavior was proportionally more often reported to improve with treatment when follow-up occurred as adults (86%, 6/7 outcomes) compared with studies of children (33%, 1/3 outcomes) and adolescents (25%, 2/8 outcomes), although this outcome may be less sytematically studied in children and adolescents [3]. A high proportion of academic outcomes were reported to benefit from treatment when follow-up occurred in children (73%, 8/11 outcomes) and adolescents (83%, 5/6 outcomes) but not for the 3 outcomes in which follow-up occurred in adults.

## Discussion

This comprehensive analysis examined 51 studies that reported 111 treatment outcomes and provided an extensive dataset for addressing questions related to the effects of specific aspects of ADHD treatment on long-term outcomes. Improvement with treatment (outcomes reported to be statistically significantly better than either pre-treatment or an untreated ADHD group) was found for all treatment modalities (pharmacological, non-pharmacological, and combination treatment) for the majority of outcomes reported. In this analysis, the comparison of treatment modalities demonstrated that the highest proportion of long-term outcomes was reported to improve with combination treatment.

The results of effect size analysis were also consistent with benefit being associated with all treatment modalities, with the largest effect sizes being associated with combination treatment. Effect sizes of individual significant improvements with treatment ranged from below the small threshold to above the large threshold for all three treatment modalities. This range of effect sizes may be expected because of the range of types of outcomes reported in these studies. All but one outcome with large treatment effect sizes were within the domains of academic, self-esteem, or social function outcomes, indicating that these areas may be particularly amenable to treatment. A smaller review by Langberg and Becker [34] of eight longitudinal studies with outcomes of ≥3 years questioned the clinical and educational significance of the magnitude of academic improvements associated with pharmacotherapy, but effect sizes were not evaluated. It is interesting that occupational outcomes, which may be considered the adult corollary of academic outcomes, were not often reported to improve with treatment and for those measures that did, the effect sizes were small. Because there were only three studies that reported occupational outcomes of treated versus untreated ADHD, further study is needed to clarify treatment effect on occupational outcomes. In addition, psychosocial interventions that specifically target occupational outcomes may need to be developed for this population. Differences observed in the response of drug use/addictive behavior may be related to the age at which the measures are taken; treatment benefit was only reported for 25% of outcomes in adolescents, but for 86% of outcomes reported in adults. Differences in drug use/addicitive behavior itself may account for the differences observed in ADHD treatment response between adolescence and adulthood. One factor may be motivation for change; adolescents are often brought to treatment by adults, whereas adults in treatment may be more self-referred.

To our knowledge, the current study is the first systematic review attempting a comprehensive comparison of treatment modalities for ADHD including a wide range of study designs and long-term functional outcomes. Many individual primary studies have compared the modalities [26,30]. One of the largest trials examining the long-term effects of different ADHD treatments on ADHD symptoms and functional outcomes was the National Institutes of Health-funded Multimodal Treatment Study of Children with *ADHD* (MTA). This randomized trial evaluated the effects of four types of treatment during a 14-month treatment period: intensive multi-component behavioral treatment, systematic pharmacological treatment, the combination of pharmacological and behavioral treatment, or routine community care [31]. For most symptoms, pharmacological treatment and combination treatment were more effective than behavioral treatment or routine community care. The results of pharmacological treatment and combination treatment were not significantly different in the primary analysis but there was non-significant rank order superiority for combination treatment over pharmacological treatment for most of the 19 symptom and outcome measures at 14 months [31]. In addition, secondary analyses reported that combined treatment was significantly better than pharmacological treatment (*d* = 0.28, *p* = 0.012) and behavioral therapy (*d* = 0.58, *p* = 0.000) in the reduction of composite symptoms and outcomes from baseline to 14 months [35,36]. This is consistent with the general pattern of effect sizes for different treatment modalities reported herein. Also observed in the MTA study was that relative efficacy of the treatment modalities differed with varying forms of comorbidity [37]. Patients with ADHD and conduct disorder/oppositional-defiant disorder had similar effect sizes with pharmacological treatment and combined treatment but minimal effect with behavioral treatment alone. Patients with ADHD and comorbid anxiety had similar effect sizes with behavioral, pharmacological, and combined treatment. Patients with ADHD and both types of comorbidities (anxiety and conduct disorder/oppositional defiant disorder) were most responsive to combination treatment.

The present study is an informative population level analysis. The choice of treatments for a given patient however, should be based on the individual’s symptoms, impairments, preferences, and the clinician’s recommendations. Nevertheless, the data suggest certain outcomes are more treatment responsive than others and that degree of benefit may also differ between treatment modalities. As more data become available, identification of the most appropriate treatment regimen for a specific patient with specific impairments may be possible.

In this analysis, shorter or longer treatment duration (≤2 years or >2 years) did not appear to have a large effect on percentage of beneficial outcomes reported, and a high proportion of beneficial outcomes were reported for each duration category. Nevertheless, because ADHD treatment duration remains a matter of debate [18] and we found a higher proportion of beneficial outcomes reported with shorter treatment duration, we examined this result further. We found that studies with a shorter duration of treatment also had a shorter time-to-follow-up (*r* = 0.50) so that the follow-up measurement was closer to treatment endpoint. Studies in the shorter follow-up time category reported a higher proportion of improved outcomes. These studies had a much shorter estimated lag time between the end of treatment and the follow-up measurement. This suggests that effects of treatment are greatest near the time when treatment has occurred, consistent with a recent review [38] that analyzed data from five randomized controlled trials, including the MTA study, and concluded that there is moderate to strong evidence for improvement in academic outcomes for follow-up times up to 14 months but that effect sizes may decrease thereafter. Thus, attenuation of effects over time after treatment cessation is a potential confound in comparison of results of studies of short and long treatment duration, because studies of short treatment duration also tend to have short follow-up times.

It is also possible that in some studies, individuals may have received additional treatment during the unmonitored follow-up period. For example, treatment modality and intensity in the MTA study were only specified during the initial 14-month randomized treatment period. The long-term follow-ups were uncontrolled, naturalistic studies with individuals and their parents free to make therapy choices. The proportion of individuals taking medication nearly converged by 3 years in contrast to the still-different rates of medication use at 2 years [27]. Qualified by these considerations, in the present analysis, the proportion of studies reporting improved outcomes was similar for longer and shorter treatment duration.

Similarily, long-term follow-up of studies using an untreated control group as the comparator may not maintain a clear untreated reference group over time; and continuation of post-study pharmacological interventions would not be subject to as frequent titration and monitoring as during the initial study period [31,34]. In addition to treatment adherence and persistence on treatment, complications of long-term follow-up studies include self-selection for continuation and development of comorbidities [32,34]. Qualified by these limitations, the current comprehensive analysis demonstrated improvement associated with treatment for 57% of outcomes reported in studies with a time to follow-up of ≥3 years from the beginning of the study.

A high proportion of beneficial outcomes were reported at all ages of treatment initiation. Most of the included studies had a mean age of treatment initiation of 8 through 12 years while fewer studies were available for the younger (<8 years) and older ages of initiation (≥13 years). Although the limited number of studies precludes a strong comparison, the proportion of studies to report benefit was similar for all ages of treatment initiation. The importance of early treatment has been previously demonstrated by within-study age comparisons. For example, a study of students in the fourth to seventh grade period [17] demonstrated less academic decline in mathematics for those starting stimulant medication at earlier ages compared to later ages in this period. The efficacy of earlier treatment is notable because early treatment initiation can be considered a marker for greater severity of ADHD; thus benefit with earlier treatment is even more impressive. On the other hand, earlier medication may also be a marker of more supportive resources. Thus, such naturalistic studies are confounded by factors such as the child’s intelligence quotient (IQ), the degree of parental support and involvement in the child’s academic progress, and other environmental factors such as socioeconomic status.

### Study limitations

A limitation of this systematic review is the inclusion of studies of widely varied characteristics, for example, different study designs, study population types and numbers, types of informant or rater, follow-up intervals, diagnostic criteria, and treatment types. In recognition of this limitation, conclusions were maintained at a general level. The purpose of this review was to include as many studies as possible that met simple, basic criteria and to provide a comprehensive summary of the included results. Nonetheless, for at least minimal criteria of quality/credibility, all studies included in the present analysis passed peer review, included a control or comparison condition, and only statistically significant improvements were counted in the present analysis as indicating benefit associated with treatment. In our assessment of the influence of study design, we found that prospective studies and RCTs showed a greater proportion of improved outcomes with treatment, compared with retrospective and cross-sectional studies. The limitations inherent to retrospective and cross-sectional studies may influence the reported outcomes. For example, self-reported historical data may be less accurate than data regarding recent or present status. Furthermore, historical data from medical and criminal records, although accurate, may not be complete (e.g., only crimes that resulted in police encounter would be in official records).

Another important limitation is that “treatment benefit” was defined as any significant improvement compared with a group of untreated individuals with ADHD *or* from pre-treatment baseline to follow-up, even without separate within-study untreated comparator groups. Many things aside from treatment, including maturation, history, placebo response, rater bias, practice effect on assessment instruments, and regression to the mean, may account for measurement of significant improvement from baseline to follow-up. Four follow-up studies of untreated individuals with ADHD compared with study-start baseline, however, reported a deterioration from baseline. Also, studies with a within-study untreated comparison group showed a similar rate of significant treatment-related improvement overall (60%). Similarly, for the few studies with a randomized untreated comparison group, the percentage of outcomes reported to improve significantly with treatment was 57% (4/7 outcomes). Finally, those who apparently got more treatment (combination of both pharmacological and nonpharmacological treatment) improved more than those who received apparently less treatment (only one type of treatment), suggesting that the amount of treatment makes a difference, and thus that treatment itself makes a difference.

There are many comorbidities associated with ADHD, including oppositional defiant disorder, conduct disorder, anxiety disorders (i.e., generalized anxiety disorder, obsessive-compulsive disorder, and posttraumatic stress disorder), and depression. The presence of comorbidities may influence the effect of treatment. Many of the studies in this dataset, however, either excluded individuals with particular comorbidities (24 of 51 studies) or controlled for comorbidities through methods such as regression analysis or comparison of outcomes of individuals with ADHD with/without the comorbidity (17 of 51 studies). Outcomes adjusted for comorbidities by some method were used in the present analysis whenever possible. The influence of comorbidites on the long-term outcomes associated with ADHD is a complex issue, especially in the consideration of the effects of treatment on these long-term outcomes. For example, this analysis showed that there were proportionally fewer studies of antisocial outcomes reporting benefit with treatment. There is evidence that, while poor antisocial outcomes appear to be associated with the presence of ADHD specifically [39,40], conduct disorder (a common comorbidity) also has a strong role in this association [19]. Thus, it may be expected that, for those individuals with comorbid ADHD and conduct disorder, treatment for ADHD specifically may not result in significant improvement in antisocial outcomes. Proper treatment and rehabilitation of such individuals is challenging [41]. Recent studies of cognitive-behavioral group therapy (the Reasoning and Rehabilitation ADHD program) either alone or in combination with pharmacological treatment, however, have reported promising results for improvement in ADHD symptoms and comorbidities [42,43]. A large Swedish study of individuals with ADHD found a significant reduction in criminality (32% for men, 41% for women) during periods on pharmacological treatment compared to periods without medication [44]. The population included patients with and without diagnosed comorbidities (conduct, oppositional-defiant, antisocial, personality, or substance-use disorder).

### Possible study biases

One bias that may occur in studies that report more than one outcome each, is that all the outcomes reported within the study may tend to be of the same type (poorer or improved; referred to as the “halo” effect), essentially giving greater weight to those studies reporting many outcomes. Analyses after collapsing all the outcomes into a single result for each study showed results similar to that presented here for the analyses of the untreated and treated outcomes, indicating that the “halo” effect was negligible for these analyses.

Another bias that may occur with the collapsing of several measures reported in a study into a single outcome group result for that study (improved or no benefit), is that for studies in which many measures were taken within a single outcome group, there is a higher likelihood of an improvement being observed for a single measure that then was summarized for the outcome group as a reported improvement (similar to the “multiple comparisons” issue in statistical comparisons). Analysis of the treated versus untreated outcomes utilizing only those outcomes stemming from consistently improved or consistently no significant improvement measures yeilded results (55% improvement associated with treatment) similar to those presented here utilizing all the outcomes reported, indicating that this effect was negligible for these analyses.

Publication bias is a potential limitation of all literature reviews, including this one. It can promote a more encouraging impression from published literature than may be gleaned from inclusion of all studies performed. The outcomes analyzed in the present analysis included every long-term outcome presented in each study, including outcomes that were reported but not necessarily the main focus of the study, thus minimizing publication bias.

## Conclusions

This comprehensive analysis demonstrated a high proportion of published long-term outcomes showing statistically significant improvement associated with each ADHD treatment modality: non-pharmacological, pharmacological, and combination treatment. Combination treatment was most consistently associated with improvement and resulted in the largest effect sizes. The most evidence for beneficial outcomes was found for self-esteem, social function, academics, and driving, and each treatment modality was associated with improvement in each of those domains. Evidence of treatment benefit was not as strong for antisocial behavior, obesity, drug use/addictive behavior, services use, or occupational outcomes, either due to a lower proportion of outcomes reported to improve with treatment or a low number of studies reporting these outcome domains. While a high proportion of long-term outcomes were reported to improve, it is possible that even the combination of current treatments are insufficient for significant improvement of a subset of specific types of outcomes, and treatments to target specific outcomes that are less responsive to current treatment options are needed.

## Supporting Information

S1 Appendix Search string. (DOCX)

S2 Appendix PRISMA checklist. (DOC)

S3 Appendix Citation list. (DOCX)

S4 Appendix Treatments identified. (DOCX)

S5 Appendix Effect sizes. (XLSX)
