# Supplementary material for: Effect of Treatment Modality on Long-Term Outcomes in Attention-Deficit/Hyperactivity Disorder: A Systematic Review
Source: PLoS One. 2015 Feb 25;10(2):e0116407. doi: 10.1371/journal.pone.0116407 (PMC4340791; doi:10.1371/journal.pone.0116407)
Supplement: S1 Appendix — (DOCX) [file pone.0116407.s001.docx]

Appendix S1

Search string and limiters

This is the actual search string and limiters copy/pasted from the search engine window.

(SU attention deficit disorder or SU hyperkine* or SU “TDAH” or SU “DAA”) and (TX "longterm" or TX "long term" or TX education or TX academic or TX degree or TX school or TX grade or TX promotion or TX "IQ" or TX “standardized test scores” or TX “standardised test scores” or TX expulsion or TX graduation or TX activities or TX sports or TX hobbies or TX socioeconomic or TX salary or TX fired or TX productivity or TX “work productivity” or TX “occupational injury” or TX emergency or TX social or TX functional or TX marriage or TX divorce or TX relations* or TX antisocial or TX criminal* or TX incarcerat* or TX arrest or TX justice or TX automobile or TX car or TX driving or TX citation* or TX crash* or TX weight or TX obesity or TX “substance use” or TX “SUD” or TX “drug abuse” or TX addicti* or TX “substance abuse” or TX “illegal substance use” or TX “illicit substance use” or TX alcoholi* or TX “alcohol abuse” or TX esteem or TX suicid*) not SU neuroanatom* not SU neuropatholog* not SU patholog* not SU pathophysiology not SU molecular not SU gene not SU genetic* not SU genotype not SU development* not SU validity not SU “differential diagnosis” not SU SPECT not SU electromyography not SU “frontal lobe” not SU “reaction time” not SU “response time” not SU "chemically induced" not SU physiopathology not SU etiology not SU aetiology not SU preclinical not SU "Phase I" not SU "Phase II" not SU tolerability not SU pharmaco* not SU dose-finding not SU modeling not SU modelling not SU ethics not SU reprint not SU “conference presentation” not SU “literature review” not PT review not PT supplement and (TX control or TX proband* or TX normal or TX placebo or TX untreated or TX “no treatment” or TX nonmedication or TX “non medication” or TX unmedicated or TX “no therapy” or TX compar* or TX premedication or TX pretreatment or TX “pre treatment” or TX longitudinal or TX retrospective or TX followup or TX “follow up” or TX “standard care”)

Limiters - Published Date from: 20110101-20111231; Peer Reviewed; Publication Year from: 2011-2011; Publication Type: Peer Reviewed Journal; English; Age Groups: Preschool Age (2-5 yrs), School Age (6-12 yrs), Adolescence (13-17 yrs), Adulthood (18 yrs & older), Young Adulthood (18-29 yrs), Thirties (30-39 yrs), Middle Age (40-64 yrs), Aged (65 yrs & older), Very Old (85 yrs & older); Population Group: Human; Intended Audience: Psychology: Professional & Research; Document Type: Chapter, Erratum/Correction, Journal Article; Methodology: EMPIRICAL STUDY, FIELD STUDY, TREATMENT OUTCOME/CLINICAL TRIAL, TWIN STUDY; Exclude Dissertations; English Language; Research Article; Clinical Queries: Therapy - High Sensitivity, Therapy - High Specificity, Therapy - Best Balance, Prognosis - High Sensitivity, Prognosis - Specificity, Prognosis - Best Balance, Qualitative - High Sensitivity, Qualitative - High Specificity, Qualitative - Best Balance; Human; Publication Type: Book Chapter, Clinical Trial, Corrected Article, Journal Article; Language: English; Age Groups: Child, Preschool: 2-5 years, Child: 6-12 years, Adolescent: 13-18 years, Adult: 19-44 years, Middle Aged: 45-64 years, Aged: 65+ years, Aged, 80 and over; Controlled Clinical Trials; MEDLINE Publication Type: Multicenter Study, Published Erratum, Randomized Controlled Trial, Twin Study, Clinical Trial, Clinical Trial, Phase III, Clinical Trial, Phase IV, Controlled Clinical Trial, Corrected and Republished Article, Journal Article; Language: English; Publication Type: Academic Journal, Book; Language: English; Document Type: Article, Book Chapter, Erratum, Report; Journal or Document: Journal Articles (EJ); Publication Type: Books, Journal Articles, Reports (All); Language: English; English Language; Human; Age Related: Child, Preschool: 2-5 years, Child: 6-12 years, Adolescent: 13-18 years, Young Adult: 19-24 years, Adult: 19-44 years, Middle Aged: 45-64 years, Middle Aged + Aged: 45 + years, Aged: 65+ years, Aged, 80 and over, All Adult: 19+ years; Clinical Queries: Therapy - High Sensitivity, Therapy - High Specificity, Therapy - Best Balance, Prognosis - High Sensitivity, Prognosis - High Specificity, Prognosis - Best Balance, Costs - High Sensitivity, Costs - High Specificity, Costs - Best Balance, Economics - High Sensitivity, Economics - High Specificity, Economics - Best Balance; Journal & Citation Subset: Core Clinical (AIM), Consumer Health, MEDLINE, Pubmed Central, PubMed not MEDLINE; Publication Type: Clinical Trial, Clinical Trial, Phase III, Clinical Trial, Phase IV, Comparative Study, Controlled Clinical Trial, Government Publications, Journal Article, Multicenter Study, Published Erratum, Randomized Controlled Trial, Twin Study; Languages: English; Publication Type: Primary Source Document, Periodical, Government Document; Publication Type: Periodical, Book; Document Type: Article, Book Chapter, Erratum, Report; Language: English

Expanders - Also search within the full text of the articles

Search modes - Boolean/Phrase
