# Supplementary material for: Effect of Treatment Modality on Long-Term Outcomes in Attention-Deficit/Hyperactivity Disorder: A Systematic Review
Source: PLoS One. 2015 Feb 25;10(2):e0116407. doi: 10.1371/journal.pone.0116407 (PMC4340791; doi:10.1371/journal.pone.0116407)
Supplement: S3 Appendix — (DOCX) [file pone.0116407.s003.docx]

Appendix S3

ADHD Long-Term Treatment Outcomes Citation Listing

1. Abikoff H, Hechtman L, Klein RG, et al. Social functioning in children with ADHD treated with long-term methylphenidate and multimodal psychosocial treatment. Journal of the American Academy of Child & Adolescent Psychiatry 2004;43(7):820-829.

2. Barkley RA, Fischer M, Smallish L, et al. Does the treatment of attention-deficit/hyperactivity disorder with stimulants contribute to drug use/abuse? A 13-year prospective study. Pediatrics 2003;111(1):97-109.

3. Biederman J, Petty CR, Fried R, et al. Educational and occupational underattainment in adults with attention-deficit/hyperactivity disorder: a controlled study. J Clin Psychiatry 2008;69(8):1217-1222.

4. Canu WH, Carlson CL. Rejection sensitivity and social outcomes of young adult men with ADHD. Journal of Attention Disorders 2007;10(3):261-275.

5. Charles L, Schain R. A four-year follow-up study of the effects of methylphenidate on the behavior and academic achievement of hyperactive children. J Abnorm Child Psychol 1981;9(4):495-505.

6. Chilcoat HD, Breslau N. Pathways from ADHD to early drug use. Journal of the American Academy of Child & Adolescent Psychiatry 1999;38(11):1347-1354.

7. Curtin C, Bandini LG, Perrin EC, et al. Prevalence of overweight in children and adolescents with attention deficit hyperactivity disorder and autism spectrum disorders: a chart review. BMC Pediatr 2005;5:48.

8. Dubnov-Raz G, Perry A, Berger I. Body mass index of children with attention-deficit/hyperactivity disorder. Journal of Child Neurology 2011;26(3):302-308.

9. DuPaul GJ, Ervin RA, Hook CL, et al. Peer tutoring for children with attention deficit hyperactivity disorder: Effects on classroom behavior and academic performance. Journal of Applied Behavior Analysis 1998;31(4):579-592.

10. Ernst M, Luckenbaugh DA, Moolchan ET, et al. Behavioral predictors of substance-use initiation in adolescents with and without attention-deficit/hyperactivity disorder. Pediatrics 2006;117(6):2030-2039.

11. Evans SW, Serpell ZN, Schultz BK, et al. Cumulative benefits of secondary school-based treatment of students with attention deficit hyperactivity disorder. School Psychology Review 2007;36(2):256-273.

12. Firestone P, Crowe D, Goodman JT, et al. Vicissitudes of follow-up studies: Differential effects of parent training and stimulant medication with hyperactives. American Journal of Orthopsychiatry 1986;56(2):184-194.

13. Fischer M, Barkley RA. Childhood stimulant treatment and risk for later substance abuse. Journal of Clinical Psychiatry 2003;64(Suppl11):19-23.

14. Fischer M, Barkley RA, Fletcher KE, et al. The stability of dimensions of behavior in ADHD and normal children over an 8-year followup. Journal of Abnormal Child Psychology: An official publication of the International Society for Research in Child and Adolescent Psychopathology 1993;21(3):315-337.

15. Frankel F, Cantwell DP, Myatt R, et al. Do stimulants improve self-esteem in children with ADHD and peer problems? Journal of Child and Adolescent Psychopharmacology 1999;9(3):185-194.

16. Frankel F, Myatt R, Cantwell DP, et al. Parent-assisted transfer of children's social skills training: Effects on children with and without attention-deficit hyperactivity disorder. Journal of the American Academy of Child & Adolescent Psychiatry 1997;36(8):1056-1064.

17. Goksøyr PK, Nøttestad JA. The burden of untreated ADHD among adults: The role of stimulant medication. Addictive Behaviors 2008;33(2):342-346.

18. Grizenko N. Outcome of multimodal day treatment for children with severe behavior problems: A five-year follow-up. Journal of the American Academy of Child & Adolescent Psychiatry 1997;36(7):989-997.

19. Halmøy A, Fasmer OB, Gillberg C, et al. Occupational outcome in adult ADHD: Impact of symptom profile, comorbid psychiatric problems, and treatment: A cross-sectional study of 414 clinically diagnosed adult ADHD patients. Journal of Attention Disorders 2009;13(2):175-187.

20. Harty SC, Ivanov I, Newcorn JH, et al. The impact of conduct disorder and stimulant medication on later substance use in an ethnically diverse sample of individuals with attention-deficit/hyperactivity disorder in childhood. Journal of Child and Adolescent Psychopharmacology 2011;21(4):331-339.

21. Hechtman L, Abikoff H, Klein RG, et al. Academic achievement and emotional status of children with ADHD treated with long-term methylphenidate and multimodal psychosocial treatment. Journal of the American Academy of Child & Adolescent Psychiatry 2004;43(7):812-819.

22. Hechtman L, Weiss G, Perlman T. Young adult outcome of hyperactive children who received long-term stimulant treatment. Journal of the American Academy of Child Psychiatry 1984;23(3):261-269.

23. Heinicke CM, Ramsey-Klee DM. Outcome of child psychotherapy as a function of frequency of session. Journal of the American Academy of Child Psychiatry 1986;25(2):247-253.

24. Huizink AC, van Lier PAC, Crijnen AAM. Attention deficit hyperactivity disorder symptoms mediate early-onset smoking. European Addiction Research 2009;15(1):1-9.

25. Huss M, Poustka F, Lehmkuhl G, et al. No increase in long-term risk for nicotine use disorders after treatment with methylphenidate in children with attention-deficit/hyperactivity disorder (ADHD): evidence from a non-randomised retrospective study. J Neural Transm 2008;115(2):335-339.

26. Jensen PS, Arnold LE, Swanson JM, et al. 3-year follow-up of the NIMH MTA study. Journal of the American Academy of Child & Adolescent Psychiatry 2007;46(8):989-1002.

27. Johnston C, Pelham WE. Teacher ratings predict peer ratings of aggression at 3-year follow-up in boys with attention deficit disorder with hyperactivity. Journal of Consulting and Clinical Psychology 1986;54(4):571-572.

28. Katusic SK, Barbaresi WJ, Colligan RC, et al. Psychostimulant treatment and risk for substance abuse among young adults with a history of attention-deficit/hyperactivity disorder: a population-based, birth cohort study. Journal of Child and Adolescent Psychopharmacology 2005;15(5):764-776.

29. Kelly PC, Cohen ML, Walker WO, et al. Self-esteem in children medically managed for attention deficit disorder. Pediatrics 1989;83(2):211-217.

30. Lambert NM, Hartsough CS, Sassone D, et al. Persistence of hyperactivity symptoms from childhood to adolescence and associated outcomes. American Journal of Orthopsychiatry 1987;57(1):22-32.

31. Leff MK, Moolchan ET, Cookus BA, et al. Predictors of smoking initiation among at risk youth: a controlled study. Journal of Child & Adolescent Substance Abuse 2003;13(1):59-75.

32. Levy LD, Fleming JP, Klar D. Treatment of refractory obesity in severely obese adults following management of newly diagnosed attention deficit hyperactivity disorder. Int J Obes (Lond) 2009;33(3):326-334.

33. Lufi D, Parish-Plass J. Sport-Based Group Therapy Program for Boys with ADHD or with Other Behavioral Disorders. Child & Family Behavior Therapy 2011;33(3):217-230.

34. Marcus SC, Wan GJ, Zhang HF, et al. Injury among stimulant-treated youth with ADHD. Journal of Attention Disorders 2008;12(1):64-69.

35. Milberger S, Biederman J, Faraone SV, et al. ADHD is associated with early initiation of cigarette smoking in children and adolescents. J Am Acad Child Adolesc Psychiatry 1997;36(1):37-44.

36. Molina BS, Hinshaw SP, Swanson JM, et al. The MTA at 8 years: prospective follow-up of children treated for combined-type ADHD in a multisite study. J Am Acad Child Adolesc Psychiatry 2009;48(5):484-500.

37. Molina BSG, Flory K, Hinshaw SP, et al. Delinquent behavior and emerging substance use in the MTA at 36 months: prevalence, course, and treatment effects. Journal of the American Academy of Child & Adolescent Psychiatry 2007;46(8):1028-1040.

38. Monuteaux MC, Spencer TJ, Faraone SV, et al. A randomized, placebo-controlled clinical trial of bupropion for the prevention of smoking in children and adolescents with attention-deficit/hyperactivity disorder. Journal of Clinical Psychiatry 2007;68(7):1094-1101.

39. Musser CJ, Ahmann PA, Theye FW, et al. Stimulant use and the potential for abuse in Wisconsin as reported by school administrators and longitudinally followed children. J Dev Behav Pediatr 1998;19(3):187-192.

40. Olfson M, Gameroff MJ, Marcus SC, et al. National trends in the treatment of attention deficit hyperactivity disorder. Am J Psychiatry 2003;160(6):1071-1077.

41. Powers RL, Marks DJ, Miller CJ, et al. Stimulant treatment in children with attention-deficit/hyperactivity disorder moderates adolescent academic outcome. Journal of Child and Adolescent Psychopharmacology 2008;18(5):449-459.

42. Satterfield JH, Satterfield BT, Cantwell DP. Three-year multimodality treatment study of 100 hyperactive boys. J Pediatr 1981;98(4):650-655.

43. Scheffler RM, Brown TT, Fulton BD, et al. Positive association between attention-deficit/hyperactivity disorder medication use and academic achievement during elementary school. Pediatrics 2009;123(5):1273-1279.

44. Schultz BK, Evans SW, Serpell ZN. Preventing failure among middle school students with attention deficit hyperactivity disorder: A survival analysis. School Psychology Review 2009;38(1):14-27.

45. Toren P, Ratner S, Weizman A, et al. Reboxetine maintenance treatment in children with attention-deficit/hyperactivity disorder: a long-term follow-up study. J Child Adolesc Psychopharmacol 2007;17(6):803-812.

46. Tymms P, Merrell C. The impact of screening and advice on inattentive, hyperactive and impulsive children. European Journal of Special Needs Education 2006;21(3):321-337.

47. Upadhyaya HP, Rose K, Wang W, et al. Attention-deficit/hyperactivity disorder, medication treatment, and substance use patterns among adolescents and young adults. Journal of Child and Adolescent Psychopharmacology 2005;15(5):799-809.

48. Vitaro F, Tremblay RE. Impact of a prevention program on aggressive children's friendships and social adjustment. Journal of Abnormal Child Psychology: An official publication of the International Society for Research in Child and Adolescent Psychopathology 1994;22(4):457-475.

49. Volpe RJ, DuPaul GJ, Jitendra AK, et al. Consultation-based academic interventions for children with attention deficit hyperactivity disorder: Effects on reading and mathematics outcomes at 1-year follow-up. School Psychology Review 2009;38(1):5-13.

50. Waldrop RD. Selection of patients for management of attention deficit hyperactivity disorder in a private practice setting. Clinical Pediatrics 1994;33(2):83-87.

51. Whalen CK, Jamner LD, Henker B, et al. Is there a link between adolescent cigarette smoking and pharmacotherapy for ADHD? Psychol Addict Behav 2003;17(4):332-335.
