# Supplementary material for: Effect of Treatment Modality on Long-Term Outcomes in Attention-Deficit/Hyperactivity Disorder: A Systematic Review
Source: PLoS One. 2015 Feb 25;10(2):e0116407. doi: 10.1371/journal.pone.0116407 (PMC4340791; doi:10.1371/journal.pone.0116407)
Supplement: S4 Appendix — (DOCX) [file pone.0116407.s004.docx]

**Appendix S4**

**Treatments Identified in Included Studies**

This list includes all the treatments mentioned in any study. Sometimes a treatment may have been listed in the Methods or Results of a study but no details were provided about dose or duration or age of treatment or frequency of treatment or separate connection to a specific outcome result, for example. It was possible to group treatment types by large category (pharmacological, non-pharmacological, or combination) and pool the reported outcomes in these categories.

Non-pharmacological Treatments: behavioral therapy, psychotherapy, family therapy, educational therapy, occupational therapy, mixed therapy (play, pet, art, drama), counseling, academic training, teacher training, parent training, social training, attention training, organizational skills training, academic mentoring, parent mentoring, diet, biofeedback.

Pharmacological Treatments: stimulants (methylphenidate, dextroamphetamine, dexamphetamine, *d*-amphetamine, lisdexamphetamine, mixed amphetamine salts, amphetamine sulfate – ‘amphetamine’ sometimes spelled ‘amfetamine’, pemoline – sometimes spelled ‘premoline’), non-stimulants (atomoxetine, reboxetine, clonidine, guanfacine), other medication including pharmaceuticals without an indication for ADHD (tricyclics, bupropion, fluoxetine, sertraline, citalopram, chlorpromazine, phenothiazine).

Combination: Any combination of the above treatments including at least one each of a pharmacological and non-pharmacological treatment.
